# Multi-Scale Transcriptomic Sequencing Data Analysis Reveals *LINC00467* is Associated with Malignant Progression in Breast Cancer: An *In Silico* and *In Vitro* Study

**DOI:** 10.32604/or.2026.067601

**Published:** 2026-03-23

**Authors:** Hui Zha, Chao Li, Jia Chen, Hao Bo, Zhaolan Hu, Zailong Qin, Jie Guo, Junbin Yuan

**Affiliations:** 1 Department of Urology, Xiangya Hospital, Central South University, Changsha, China; 2 NHC Key Laboratory of Human Stem Cell and Reproductive Engineering, Institute of Reproductive and Stem Cell Engineering, Central South University, Changsha, China; 3 Department of Plastic Surgery, The Third Xiangya Hospital, Central South University, Changsha, China; 4 Department of Anesthesiology, The Second Xiangya Hospital, Central South University, Changsha, China; 5 Guangxi Key Laboratory of Reproductive Health and Birth Defect Prevention, Maternal and Child Health Hospital of Guangxi Zhuang Autonomous Region, Nanning, China; 6 Genetic and Metabolic Central Laboratory, Birth Defects Prevention and Control Institute of Guangxi Zhuang Autonomous Region, Nanning, China; 7 National Institute of Drug Clinical Trial, Xiangya Hospital, Central South University, Changsha, China; 8 China National Clinical Research Center for Geriatric Disorders, Xiangya Hospital, Central South University, Changsha, China

**Keywords:** Breast cancer, *LINC00467*, cell stemness, single cell sequencing, drug sensitivity

## Abstract

**Objective:**

Long non-coding RNAs have been found to play a pivotal role in breast cancer, yet the majority of these lncRNAs remain to be thoroughly investigated. This study aimed to explore the role of differentially expressed long non-coding RNAs (lncRNAs) in breast cancer stemness and drug sensitivity.

**Methods:**

Database mining was performed to evaluate the expression of *LINC00467* in different types of breast cancer and its association with clinical features. The function of *LINC00467* was examined through colony formation assays, quantitative reverse transcription PCR (qRT-PCR), and western blotting following *LINC00467* silencing in breast cancer cell lines.

**Results:**

*LINC00467* was significantly upregulated in various breast cancer subtypes with spatial specificity. Silencing *LINC00467* reduced clonogenic capacity and downregulated the stemness-associated factor *LIN28B* as well as phosphorylated RAC-alpha serine/threonine-protein kinase (p-AKT). The transcription factors specificity protein 1 (*SP1*) and E2F transcription factor 1 (*E2F1*) were predicted to bind to the *LINC00467* promoter. Furthermore, breast cancer samples with high *LINC00467* expression displayed reduced sensitivity to AKT inhibitors, and high *LINC00467* expression was negatively correlated with the therapeutic response to programmed cell death 1 (PD-1) antibodies.

**Conclusion:**

Our findings suggest that spatially expressed *LINC00467* may promote breast cancer stemness by regulating AKT signaling and could serve as a potential new therapeutic target and indicator of drug sensitivity in breast cancer.

## 1 Introduction

Breast cancer is one of the most common malignant tumors in women, and its incidence continues to rise worldwide [1]. According to global cancer statistics, breast cancer accounts for 25% of all cancer cases and 15% of cancer-related deaths [2]. Current treatment strategies typically involve a combination of hormonal therapy, surgery, chemotherapy, and radiotherapy. However, prognosis remains poor due to multidrug resistance, recurrence, and metastasis. Consequently, identifying specific biomarkers and therapeutic targets is critical for the early diagnosis and effective treatment of breast cancer [3].

Long non-coding RNAs (lncRNAs) are a class of RNA molecules longer than 200 nucleotides that lack protein-coding potential and are generally poorly conserved [4]. They regulate diverse biological processes through mechanisms such as anchoring transcription factors, modulating alternative splicing, activating microRNAs, and recruiting chromatin-modifying enzymes [5]. In recent years, lncRNAs have received increasing attention because of their crucial roles in various human diseases, including breast cancer [6]. Several studies have found that lncRNAs are aberrantly expressed in a variety of cancers, including breast cancer [7]. Such dysregulated lncRNAs may serve as valuable biomarkers for cancer screening and diagnosis [8,9]. Moreover, their altered expression can influence tumor progression and chemotherapy resistance [10,11]. For example, *LINC00467*, a testis-specific lncRNA, has been shown to reduce viability and promote apoptosis when silenced in adult neuroblastoma cells [12,13]. Consistent with these findings, our previous study demonstrated that elevated *LINC00467* expression was associated with poorer overall survival in breast cancer patients. Furthermore, we revealed that *LINC00467* promotes breast cancer progression by directly interacting with miR-138-5p and *LIN28B* [14]. However, the utilization of spatial transcriptomics and single-cell transcriptome data remains relatively low, with a vast amount of spatially specific gene expression and function yet to be elucidated. The role of lncRNAs in the spatial heterogeneity of breast cancer is almost entirely unexplored. In this study, we integrated spatial transcriptomics, single-cell RNA sequencing, and bulk RNA sequencing data for analysis and identified a spatially highly variable lncRNA—*LINC00467*—with important functions in breast cancer.

The pronounced heterogeneity and stemness of breast cancer cells contribute to their low sensitivity to therapeutic drugs [15]. In recent years, spatial transcriptomics and single-cell transcriptomics have become important approaches for elucidating the pathogenesis of breast cancer [16]. However, their application remains limited, and the expression and function of many spatially specific genes are still poorly characterized. In particular, the role of lncRNAs in the spatial heterogeneity of breast cancer remains largely unexplored.

In this study, we integrated spatial transcriptomic, single-cell sequencing, and bulk RNA sequencing data to identify *LINC00467*, a spatially highly variable lncRNA with potential functional significance in breast cancer. However, its relationship with breast cancer stemness and drug sensitivity has not been fully clarified. Here, we conducted cell experiments, molecular biology experiments and a series of bioinformatics analysis to explore the role of *LINC00467* in breast cancer prognosis and drug treatment response.

## 2 Materials and Methods

### 2.1 Data Mining

Spatial transcriptomic and single-cell sequencing data from breast cancer patients were downloaded from STOmicsDB (https://db.cngb.org/stomics/), the spatial resolution of the spatial transcriptome chip is 55 μm. Venny 2.1 online tool (https://bioinfogp.cnb.csic.es/tools/venny/index.html) was used to draw the venn diagram. Clustering analysis was performed in R (v4.2.1) using the ‘Seurat’ package (v4.2.3). Specifically, the *FindClusters* function was applied to the spatial transcriptomic data, and each cluster was classified and labeled as either “Tumor” or “Non-Tumor” based on corresponding pathological images. The *VlnPlot* function was then used to visualize the expression of target lncRNAs across clusters. Spatially specific genes were identified using SpatialDE (https://github.com/Teichlab/SpatialDE) with default parameters. Genes with the lowest q-values were intersected with lncRNAs annotated in the Ensembl database (https://www.ensembl.org/index.html?redirect=no) [17], yielding 10 spatially specific lncRNAs. These 10 candidates were further intersected with lncRNAs significantly differentially expressed in the TCGA breast cancer (BRCA) cohort available in GEPIA2 (|log2FC| > 1, *p* < 0.05), and the final target molecules were obtained. Using the TCGA BRCA cohort data from the BEST online database (https://rookieutopia.hiplot.com.cn/app_direct/BEST/), samples were stratified into high- and low-expression groups based on the median expression level of *LINC00467*. Gene Set Enrichment Analysis (GSEA) and mapping of the top 20 high-frequency mutated genes were then performed. Overall survival (OS) curves were generated using the Kaplan-Meier Plotter online tool ( http://kmplot.com/analysis/index.php?p=service) [18]. Expression levels of *LINC00467* across the four breast cancer subtypes, as well as corresponding image mapping, were analyzed using GEPIA2 (http://gepia2.cancer-pku.cn/#index) [19], based on the TCGA BRCA cohort data. The correlation between *LINC00467* expression and *ER*, *KI67*, *PR* positive and treatment methods in breast cancer patients was analyzed using the dataset GSE21653 and GSE9893 [20,21]. The single-cell RNA sequencing dataset GSE212461 was used to analyze the expression pattern of *LINC00467* in different breast cancer cell subclusters [22]. Finally, correlations between *LINC00467* and *ALDH2, ALDH6A1, CD44, LIN28B, SP1*, and *E2F1* were assessed using GEPIA2. Associations between *LINC00467* and tumor-associated signaling pathways in breast cancer were analyzed through GSCA (https://guolab.wchscu.cn/GSCA/#/) based on TCGA BRCA cohort data [23]. The competitive endogenous RNA (ceRNA) regulatory network analysis of *LINC00467* was performed using the RNAInter online database (http://www.rnainter.org/) [24]. AnimalTFDB (https://guolab.wchscu.cn/AnimalTFDB4/#/) [25], RegNetwork (http://www.regnetworkweb.org/search.jsp) [26], and PROMO (http://alggen.lsi.upc.es/cgi-bin/promo_v3/promo/promoinit.cgi?dirDB=TF_8.3) [27] online tools were used to predict the transcription factors of *LINC00467*, and finally take the intersection of the prediction results of the three databases. We used the chromatin immunoprecipitation seq (chip-seq) data of *SP1* and *E2F1* of MCF-7 and MDA-MB-231 cells in the UCSC database [28] to analyze the binding of these two transcription factors in the promoter region of *LINC00467*.

### 2.2 Cell Culture and Transfection

All cell lines (MCF-7 and MDA-MB-231) used in this study were purchased from Wuhan Procell Company, authenticated by short tandem repeat (STR) profiling, and confirmed to be free of mycoplasma contamination. Cells were cultured in Dulbecco’s Modified Eagle’s Medium (Thermo Fisher Scientific, cat#11960044, Waltham, MA, USA) containing 10% fetal bovine serum (Thermo Fisher Scientific, cat#A5670801, Waltham, MA, USA), 100 U/mL penicillin, and 100 µg/mL streptomycin (Thermo Fisher Scientific, cat#15140122, Waltham, MA, USA). Cultures were maintained at 37°C in a humidified atmosphere with 5% CO_2_. Plasmid transfection was carried out using the Lipofectamine 3000 Transfection Kit (Thermo Fisher Scientific, cat#L3000015, Waltham, MA, USA) following the manufacturer’s instructions. Small interfering RNA (siRNA) sequences targeting *LINC00467* were synthesized by RiboBio (Guangzhou, China, cat#siB000000). The sequence used was: LINC00470-siRNA: CTGAGTTGCAGAAACAAAT.

### 2.3 Quantitative Real-Time PCR

MCF-7 and MDA-MB-231 cells were washed three times with pre-cooled phosphate-buffered saline (1× PBS, pH = 7.4), and total RNA was extracted using TRIzol reagent (2 mL TRIzol per 60 mm dish; Thermo Fisher Scientific, cat#10296028CN, Waltham, MA, USA). A 1–2 μL aliquot was used to assess RNA concentration and quality at wavelengths of 260 and 280 nm with a spectrophotometer (Thermo Fisher Scientific, Waltham, MA), and the remaining RNA was stored at –80°C. The expression of *LINC00467* and *LIN28B* was quantified by real-time fluorescence quantitative polymerase chain reaction (qPCR) using the LightCycler 480 system (Roche, Basel, Switzerland), with β-actin serving as the internal control. Primers were designed and synthesized by Shanghai Biotechnology Co., Ltd., with the following sequences: *LINC00467*-F: 5^′^-TCGTCTTCAGGAAGCCAGAC-3^′^; *LINC00467*-R: 5^′^-TGGAAATCAAAAGGGTCAGC-3^′^; *LIN28B*-F: 5^′^-CACGAGTTTGGAGCTGAGGG-3^′^; *LIN28B*-R: 5^′^-AGGTAGACTTTGCAACCGGG-3^′^; β-actin-F: 5^′^-TCACCAACTGGGACGACATG-3^′^; β-actin-R: 5^′^-GTCACCGGAGTCCATCACGAT-3^′^.

### 2.4 Clone Formation

Digested MCF-7 and MDA-MB-231 cells were resuspended in culture medium, and cell numbers were determined using a hemocytometer (Hausser Scientific, Horsham, PA, USA) under the 1 mm × 1 mm × 0.1 mm grid counting mode. Cells were seeded at a density of 1000 cells per well for colony formation assays. After 10 days of incubation, colonies were washed twice with 1× PBS (pH = 7.4), stained with 1% crystal violet for 15 min, and photographed for colony counting.

### 2.5 Western Blot

Protein blotting was performed as previously described [29]. Briefly, total protein was extracted from MCF-7 cells using RIPA lysis buffer (Beyotime, cat# P0038, Beijing, China), and protein concentrations were determined with the BCA Protein Assay Kit (Thermo Fisher Scientific, cat#A65453, Waltham, MA, USA). Equal amounts (50 μg) of protein were separated on 10% SDS-PAGE gels and transferred onto polyvinylidene difluoride (PVDF) membranes. Membranes were blocked with non-fat powdered milk (Beyotime, cat# P0216-300g, Beijing, China) at room temperature for 1 h and then incubated overnight at 4°C with the following primary antibodies: anti-AKT (1:1000, Cell Signaling Technology, cat#9272, Danvers, MA, USA), anti-p-AKT (1:1000, Cell Signaling Technology, cat#4060, Danvers, MA, USA), and monoclonal mouse anti-GAPDH (1:3000, ProMab Biotechnologies, cat#20035, Changsha, China). After washing, membranes were incubated with the appropriate secondary antibody (1:2000, Cell Signaling Technology, cat#7076, Danvers, MA, USA) for 1 h at room temperature. Protein bands were visualized using an enhanced chemiluminescence (ECL) detection kit (Millipore, cat#WBKLS0500, Darmstadt, Germany). Each experiment was repeated three times.

### 2.6 Statistical Analysis

Statistical analyses were performed using GraphPad Prism 9 software (GraphPad Software, San Diego, CA, USA). Data are presented as mean ± SEM. Comparisons between two groups were conducted using Student’s *t*-test or Wilcoxon test. Correlation analysis was performed using Spearman correlation analysis. Survival analysis was performed using the log-rank test. And *p* < 0.05 was considered statistically significant.

## 3 Results

### 3.1 The Expression of LINC00467 in Breast Cancer and Its Correlation with the Clinicopathological Characteristics of Patients with Breast Cancer

**Figure 1:**
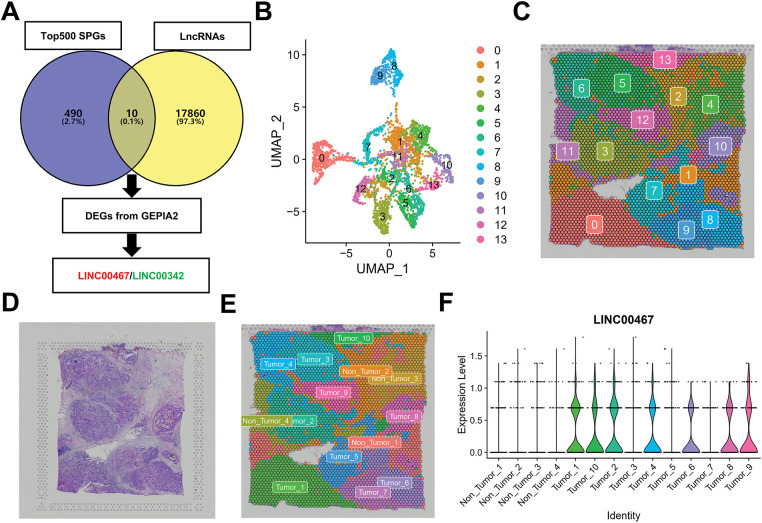

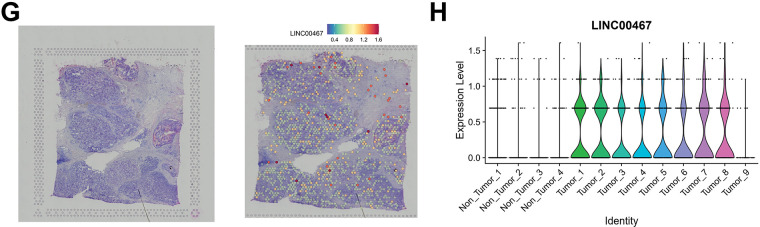
Screening of spatially hypervariable lncRNAs. (**A**) Workflow for identifying breast cancer spatially hypervariable lncRNAs: the top 500 spatially variable genes from STOmicsDB were intersected with lncRNAs from the Ensembl database, and further filtered using GEPIA2, yielding *LINC00467* and *LINC00342*. (**B**,**C**) Downscaling clustering analysis of spatial transcriptome data using the ‘Seurat’ package identified 13 subgroups. (**D**) Hematoxylin and eosin (H&E) staining of the corresponding patient section. (**E**) Pathologist’s annotation of each subgroup. (**F**) Expression of *LINC00467* across subgroups (Section 1), showing minimal expression in non-tumor tissues and high expression in tumor tissues. (**G**) Analysis of an additional breast cancer section from STOmicsDB confirmed consistent expression and spatial localization of *LINC00467*. (**H**) The expression level of *LINC00467* in each subgroup of Section 2.

**Figure A1:**
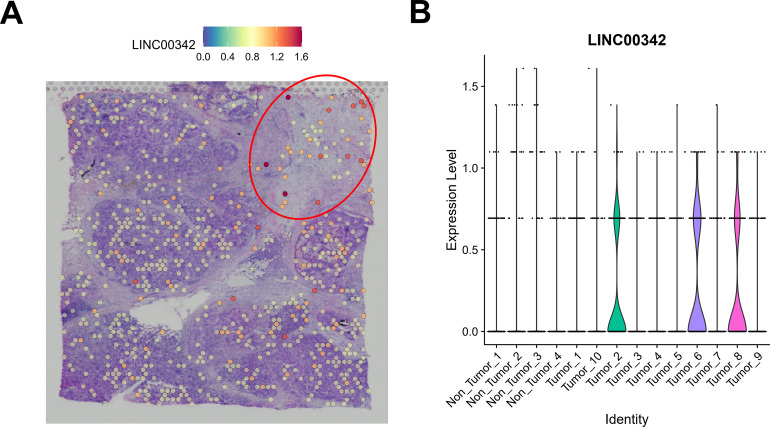
Spatial expression characteristics of *LINC00342*. (**A**) Spatial expression of *LINC00342* in breast cancer samples, showing lower expression positivity compared with *LINC00467*. Red circles indicate that *LINC00342* is also expressed in non-tumor regions. (**B**) Violin plot showing the distribution of *LINC00342* expression across the different subgroups.

To identify spatially highly variable lncRNAs associated with breast cancer, we first downloaded the top 500 candidates from the STOmicsDB spatial transcriptome database and intersected them with lncRNAs annotated in the Ensembl database [30]. Differentially expressed lncRNAs in breast cancer were further filtered using the GEPIA2 database [19]. This analysis yielded two breast cancer–associated spatially highly variable lncRNAs, *LINC00467* and *LINC00342* (Fig. 1A). Downscaling clustering of the STOmicsDB spatial transcriptome data using the ‘Seurat’ package in R identified 13 subgroups (Fig. 1B,C). Pathologist-annotated Ensembl markers were then used to characterize each subgroup (Fig. 1D,E). We observed marked heterogeneity in *LINC00467* expression across subgroups: it was nearly absent in non-tumor tissues but strongly expressed in tumor tissues (Fig. 1F). Analysis of an additional slice from the same patient confirmed the consistent expression pattern and spatial localization of *LINC00467* in breast cancer (Fig. 1G,H). However, *LINC00342* showed a lower positive rate and weaker expression in breast cancer samples compared with *LINC00467*, and it was also detected in non-tumor regions (highlighted by red circles) (Fig. A1). Based on these findings, *LINC00467* was selected for subsequent analyses.

**Figure 2:**
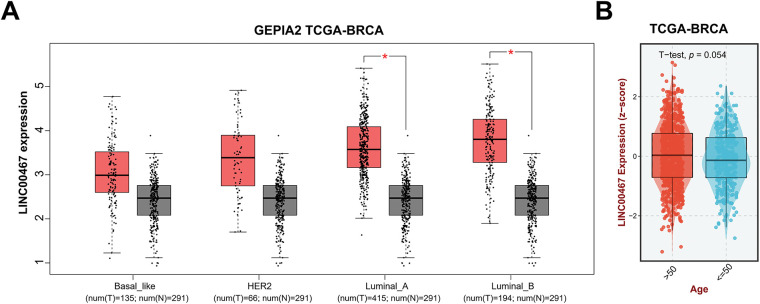

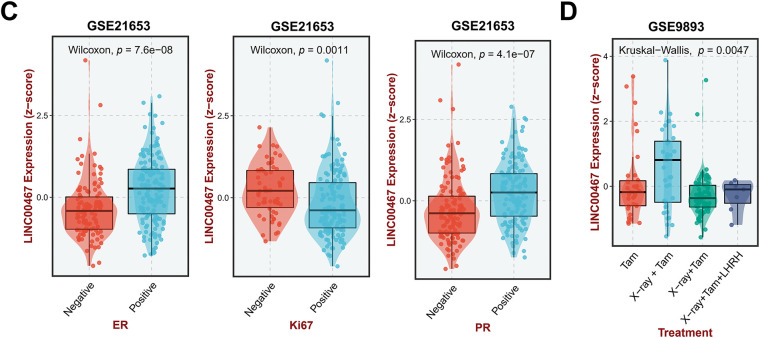
Correlation of *LINC00467* expression with clinicopathological characteristics of breast cancer patients. (**A**) *LINC00467* expression was significantly upregulated in Luminal A and Luminal B subtypes, with a slight but non-significant increase in Basal-like and HER2 subtypes. (**B**) *LINC00467* expression showed no correlation with patient age. (**C**) *LINC00467* expression was higher in estrogen receptor (ER)-positive and progesterone receptor (PR)-positive breast cancer patients but lower in *Ki67*-positive patients. (**D**) *LINC00467* expression varied across treatment regimens and was significantly higher in patients treated with X-ray compared with those treated with luteinizing hormone-releasing hormone (LHRH) or tamoxifen (Tam). *p < 0.05.

We used the GEPIA2 database to analyze the correlation between *LINC00467* expression and the clinicopathological characteristics of breast cancer patients. *LINC00467* was significantly upregulated in Luminal A and Luminal B subtypes and showed a slight, non-significant increase in Basal-like and HER2 subtypes (Fig. 2A). Its expression was not associated with the age of breast cancer adenocarcinoma patients (Fig. 2B). However, *LINC00467* expression was positively correlated with estrogen receptor (ER) and progesterone receptor (PR) expression but negatively correlated with the proliferation marker *Ki67* (Fig. 2C). Furthermore, *LINC00467* expression varied according to treatment regimen, being significantly higher in patients treated with X-ray compared with those treated with tamoxifen (Fig. 2D).

**Figure 3:**
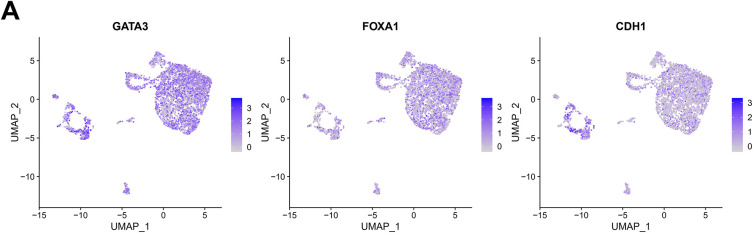

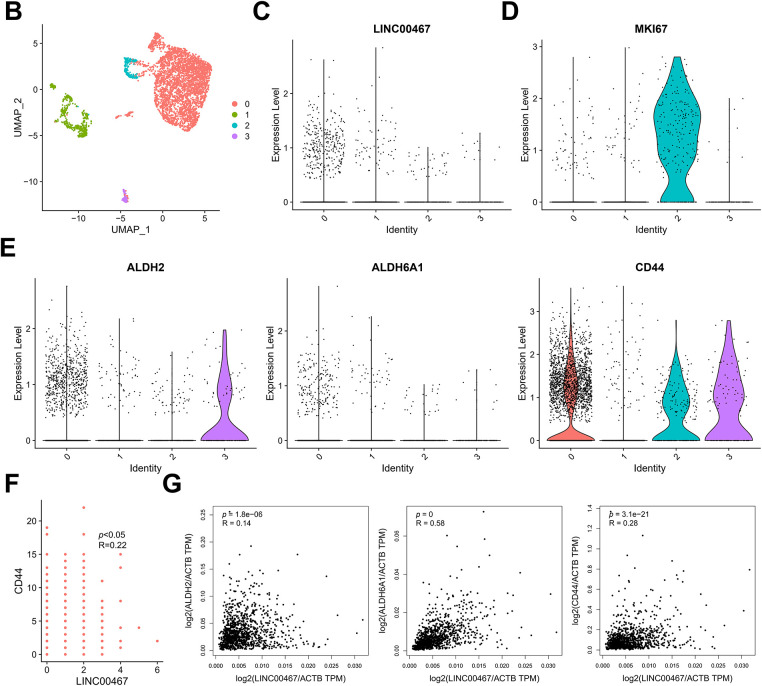
*LINC00467* is associated with stemness in breast cancer. (**A**) Marker gene display of breast cancer cells in the GSE212461 dataset. (**B**) Tumor cells in the GSE212461 dataset were downscaled into 4 subpopulations. (**C**) *LINC00467* showed higher expression in subpopulation 0. (**D**) Proliferation marker *MKI67* was highly expressed in subpopulation 2. (**E**) The breast cancer stemness-associated marker was highly expressed in subpopulation 0. (**F**) Spatial transcriptomics analysis identified a significant positive correlation between *LINC00467* and the stem cell marker *CD44* at the gene expression level. (**G**) Validation of the positive correlation between *LINC00467* and stemness-associated markers (*ALDH2*, *ALDH6A1*, and *CD44*) using TCGA BRCA data.3.2 Silencing of *LINC00467* significantly inhibited clone formation in breast cancer cells as well as cancer cell stemness.

We further integrated single-cell sequencing and the TCGA data to analyze the correlation between *LINC00467* and tumor cell stemness in breast cancer patients. Tumor cells from the GSE212461 dataset were downscaled into four subgroups (Fig. 3A,B). *LINC00467* showed low expression in subgroup 2 and high expression in subgroup 0. Proliferation and stemness analysis revealed that the proliferation marker *MKI67* was highly expressed in subgroup 2, whereas breast cancer stemness-related markers were highly expressed in subgroup 0 (Fig. 3C–E). To further substantiate their relationship, we examined the spatial co-localization of *LINC00467* with stem cell markers (e.g., *CD44*). The data demonstrated a positive correlation between *LINC00467* and the stem cell marker *CD44* at the gene expression level (Fig. 3F). The positive correlation between *LINC00467* and stemness-related markers (*ALDH2*, *ALDH6A1*, and *CD44*) was further confirmed using TCGA BRCA data (Fig. 3G). Collectively, these findings suggest that high *LINC00467* expression represents a poor prognostic factor in breast cancer and may contribute to maintaining tumor cell stemness.

### 3.2 Silencing of LINC00467 Significantly Inhibited Clone Formation in Breast Cancer Cells as Well as Cancer Cell Stemness

**Figure 4:**
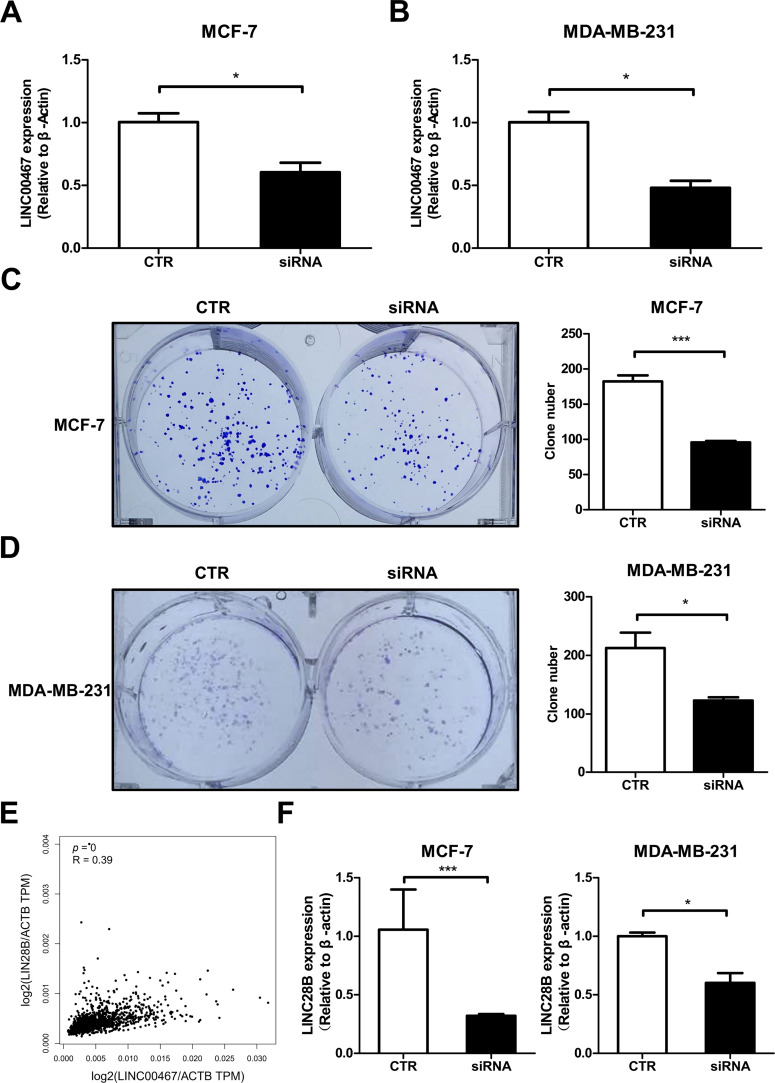
*LINC00467* correlates with clonogenic ability and stemness genes in breast cancer. (**A**,**B**) qRT-PCR detection of *LINC00467* expression in MCF-7 and MDA-MB-231 cells after transfection with *LINC00467* siRNA, CTR, control.(**C**,**D**) Silencing of *LINC00467* significantly inhibits breast cancer cell clonogenic ability. (**E**) TCGA BRCA cohort analysis showing a significant positive correlation between *LINC00467* and *LIN28B* expression. (**F**) qRT-PCR analysis of *LIN28B* expression after silencing *LINC00467*. Knockdown of *LINC00467* significantly suppressed *LIN28B* expression. *p < 0.05, ***p < 0.001.

**Figure A2:**
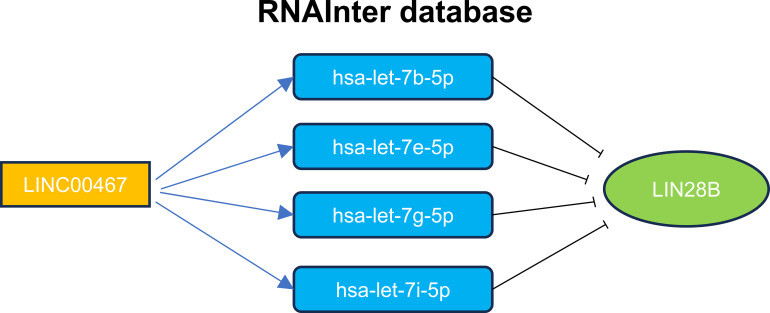
This model proposes that *LINC00467* functions as a molecular sponge, sequestering let-7 family miRNAs and thereby potentially upregulating *LIN28B* to promote cellular stemness.

To further investigate the function of *LINC00467* in breast cancer, we designed siRNA specifically targeting *LINC00467*. Following transfection in both MCF-7 and MDA-MB-231 cells, *LINC00467* expression was significantly downregulated (Fig. 4A,B). Colony formation assays revealed that silencing *LINC00467* markedly reduced the clonogenic capacity of both MCF-7 and MDA-MB-231 cells (Fig. 4C,D). In an effort to elucidate the functional partners of *LINC00467*, we conducted computational screening using the RNAInter database (http://www.rnainter.org/). The analysis revealed that *LINC00467* may interact with several let-7 family miRNAs, including hsa-let-7b-5p, hsa-let-7e-5p, hsa-let-7g-5p, and hsa-let-7i-5p [24]. Given that let-7 family miRNAs have been well-established as negative regulators targeting *LIN28B* [31], a key factor in modulating cellular stemness, we propose that *LINC00467* may function as a molecular sponge for let-7 miRNAs, thereby alleviating their inhibitory effect on *LIN28B*. (Fig. A2). Given that the multipotency factor *LIN28B* is known to promote stemness in breast cancer cells, we next analyzed data from the TCGA BRCA cohort and identified a significant positive correlation between *LINC00467* and *LIN28B* expression (Fig. 4E). Consistently, knockdown of *LINC00467* significantly suppressed *LIN28B* expression (Fig. 4F), suggesting that *LINC00467* may regulate breast cancer stemness, at least in part, through *LIN28B*.

### 3.3 LINC00467 Alters AKT Signaling and Correlates with Drug Treatment Sensitivity

**Figure 5:**
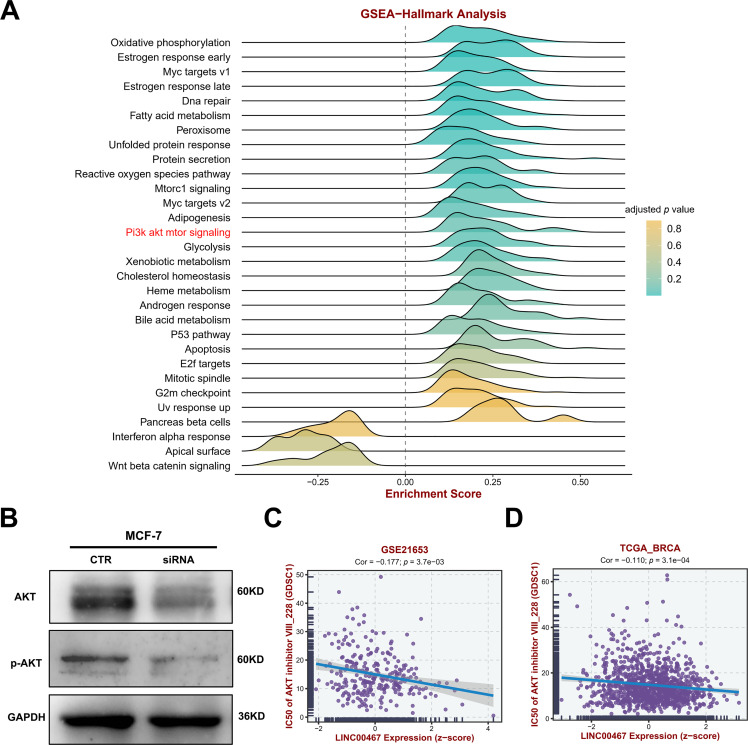
*LINC00467* regulates AKT signaling and correlates with AKT-related drug sensitivity. (**A**) GSEA enrichment analysis using TCGA BRCA cohort data from the BEST online database, dividing samples into two groups of high and low *LINC00467* expression based on median expression, showing that *LINC00467* was significantly associated with AKT signaling. (**B**) Silencing of *LINC00467* significantly inhibited AKT expression. (**C**,**D**) Analysis of the relationship between *LINC00467* expression and AKT-related drug sensitivity using GSE21653 and TCGA BRCA data from the BEST online tool. *LINC00467* expression was significantly negatively correlated with AKT-related drug sensitivity.

**Figure 6:**
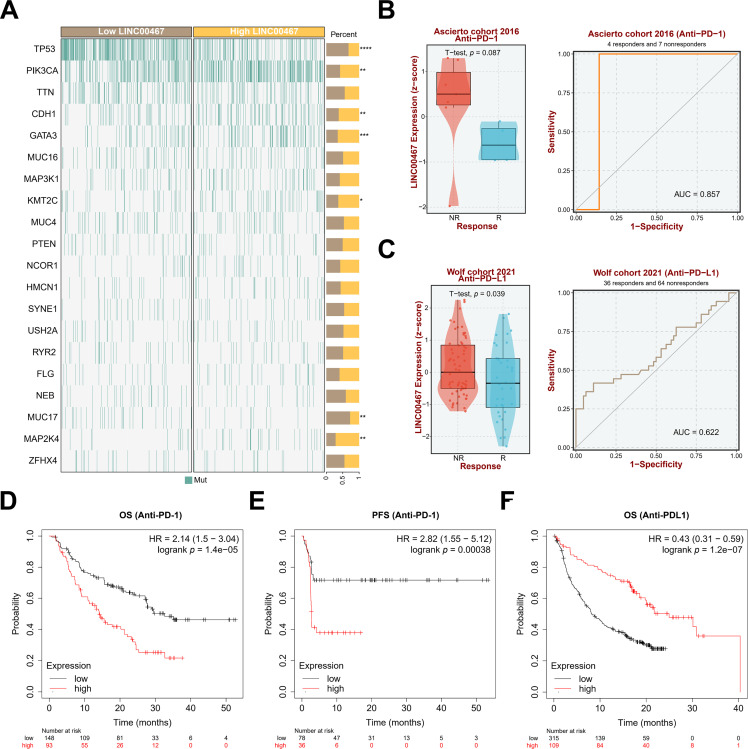
Mutation profiles in high- and low-*LINC00467* expression groups and their relationship with immunotherapy response in tumor patients. (**A**) TCGA BRCA cohort data from the BEST online database were stratified into high- and low-*LINC00467* expression groups to generate the top 20 most frequently mutated genes. TP53 showed a lower mutation frequency in the high-*LINC00467* group, whereas *PIK3CA* and *CDH1* mutations were more frequent. (**B**) *LINC00467* expression was significantly higher in the non-responsive (NR) group compared with the responsive (R) group of patients receiving anti-PD-1 immunotherapy. (**C**) Elevated *LINC00467* expression was associated with worse prognosis among patients treated with anti-PD-1 immunotherapy. (**D**) The correlation between the expression level of *LINC00467* and the overall survival of patients treated with anti-PD-1. (**E**) The correlation between the expression level of *LINC00467* and the progression-free survival of patients treated with anti-PD-1. (**F**) The correlation between the expression level of *LINC00467* and the overall survival of patients treated with anti-PDL-1. *p < 0.05, **p < 0.01, ***p < 0.001, ****p < 0.0001.

**Figure A3:**
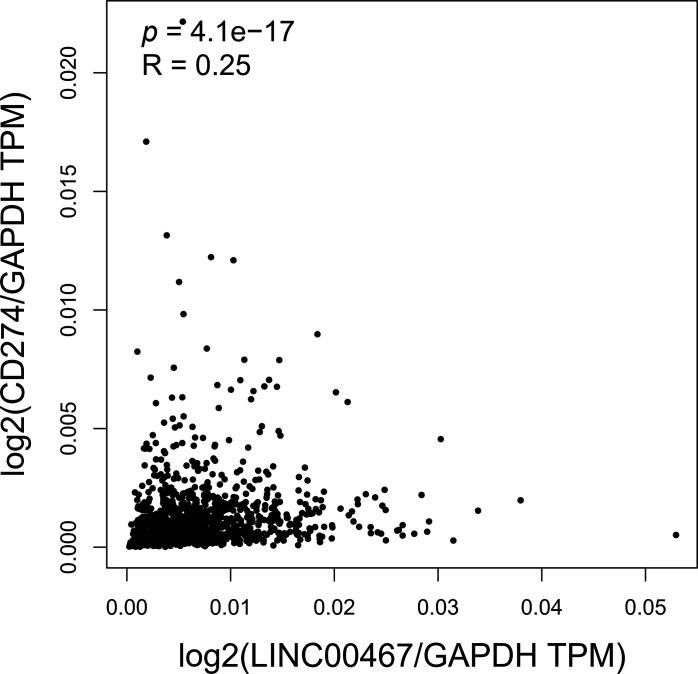
Analysis of the BRCA dataset from the GEPIA2 database reveals a significant positive correlation between *LINC00467* and *CD274* (PD-L1) expression.

To further explore the downstream signaling pathways regulated by *LINC00467*, we analyzed its association with tumor-related pathways using the TCGA BRCA cohort data. *LINC00467* expression showed a significant positive correlation with the PI3K/AKT pathway (Fig. 5A). Consistently, silencing *LINC00467* markedly reduced both total AKT and phosphorylated AKT protein levels (Fig. 5B). Moreover, breast cancer cells with high *LINC00467* expression exhibited increased sensitivity to AKT inhibitors compared with those with low *LINC00467* expression (Fig. 5C,D). We further examined the mutational landscape and its relationship with immunotherapeutic response in patients stratified by *LINC00467* expression. Using TCGA BRCA cohort data from the BEST online database, samples were divided into high- and low-expression groups based on the median *LINC00467* expression level. The top 20 most frequently mutated genes revealed that *TP53* mutations were less common in the high-expression group, whereas *PIK3CA* mutations were more prevalent (Fig. 6A). In addition, *LINC00467* expression was significantly higher in patients who were non-responsive (NR) to anti-PD-1 immunotherapy compared with responders, suggesting that *LINC00467* may serve as a predictive marker of immunotherapy efficacy (Fig. 6B,C). It is noteworthy that higher expression of *LINC00467* correlates with poorer prognosis in patients receiving anti-PD-1 therapy, whereas it is associated with better prognosis in those treated with anti-PD-L1 therapy. (Fig. 6D–F). We performed a correlation analysis using the publicly available BRCA dataset from the GEPIA2 database. The results demonstrated a significant positive correlation between the expression of *LINC00467* and *CD274* (*PD-L1*) (Fig. A3).

### 3.4 SP1 and E2F1 May be the Regulatory Transcription Factors of LINC00467

**Figure 7:**
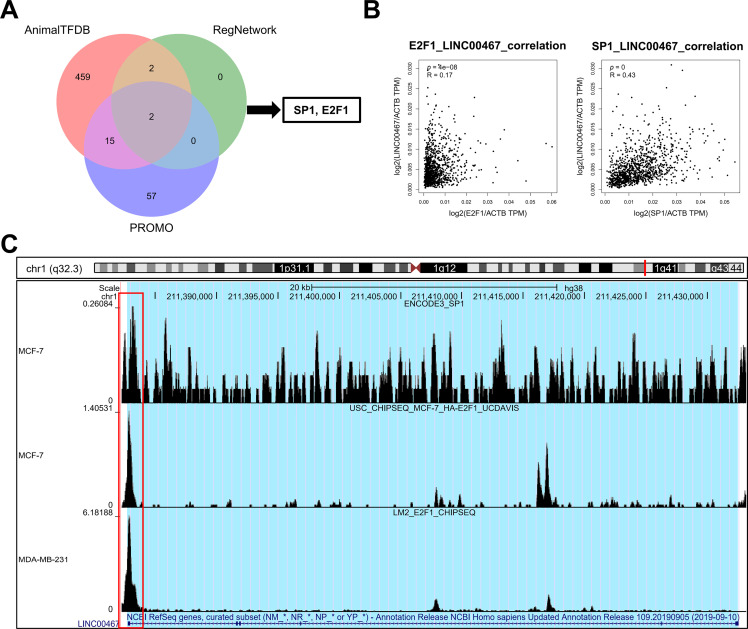
Prediction and validation of transcription factors regulating *LINC00467*. (**A**) Transcription factors of *LINC00467* were predicted using three databases (AnimalTFDB, RegNetwork, and PROMO), with *E2F1* and *SP1* identified as common candidates. (**B**) Correlation analysis showed that *LINC00467* expression was positively associated with *E2F1* and *SP1* expression. (**C**) Chip-seq data revealed significant binding peaks for *E2F1* and *SP1* near the *LINC00467* promoter region, suggesting direct transcriptional regulation.

**Figure A4:**
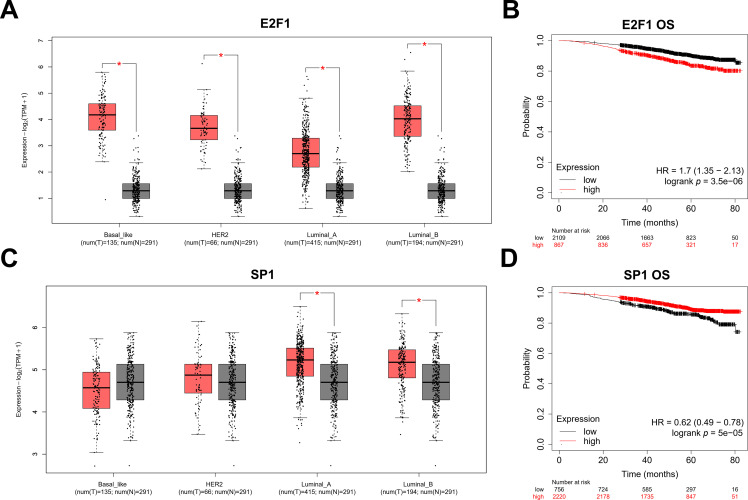
Expression of transcription factors *E2F1* and *SP1* in breast cancer and their correlation with prognosis. (**A**) *E2F1* expression was significantly upregulated in Luminal A, Luminal B, Basal-like, and HER2 subtypes. (**B**) Kaplan–Meier survival analysis based on TCGA BRCA data, stratified by median *E2F1* expression, showed that high *E2F1* expression was associated with reduced overall survival. (**C**) *SP1* expression was significantly upregulated in Luminal A and Luminal B breast cancer tissues compared to normal tissues. In contrast, there was no significant difference in expression in Basal-like and HER2 subtypes. (**D**) The samples were divided into two groups based on the median level of *SP1* expression, and in the group with high *SP1* expression, the survival rate was higher. *p < 0.05.

To explore the upstream transcriptional regulation of *LINC00467*, three transcription factor prediction tools—AnimalTFDB (https://guolab.wchscu.cn/AnimalTFDB4/#/), RegNetwork (http://www.regnetworkweb.org/search.jsp), and PROMO (http://alggen.lsi.upc.es/cgi-bin/promo_v3/promo/promoinit.cgi?dirDB=TF_8.3)—were used. The intersection of predictions from these databases identified two potential transcription factors, *SP1* and *E2F1* (Fig. 7A). *LINC00467* expression was positively correlated with both factors (Fig. 7B). Analysis of publicly available ChIP-seq data revealed significant peaks for *E2F1* and *SP1* in the promoter region of *LINC00467* (Fig. 7C), suggesting that these transcription factors may directly regulate its expression. *E2F1* expression was significantly upregulated in Luminal A, Luminal B, Basal-like, and HER2 breast cancer subtypes (Fig. A4A). Stratification of patients into high- and low-*E2F1* groups based on median expression showed that higher *E2F1* expression was associated with poorer overall survival (Fig. A4B). In contrast, *SP1* expression was significantly elevated in Luminal A and Luminal B breast cancer tissues compared with normal tissues but showed no significant difference in Basal-like or HER2 subtypes (Fig. A4C). Notably, higher *SP1* expression was associated with better survival (Fig. A4D).

## 4 Discussion

The intra-tumoral heterogeneity (ITH) theory differs from the molecular typing theory of breast cancer. ITH refers to the coexistence of multiple subtypes within the same tumor (spatial heterogeneity) and the dynamic interconversion between phenotypes over time (temporal heterogeneity) [32]. Single-cell RNA sequencing (scRNA-seq) enables detailed characterization of cell states and population heterogeneity, thereby providing insights into the mechanisms underlying breast cancer progression and metastasis, and offering a foundation for individualized therapy [33]. Using scRNA-seq, researchers have characterized tumor heterogeneity across different molecular subtypes and identified specific cell populations linked to poor prognosis or therapeutic response [34]. Single-cell profiling of the breast cancer tumor microenvironment has revealed immune cell subpopulations that may be potential targets for immunotherapy [35]. In addition, scRNA-seq can be applied to investigate intercellular signaling, regulatory single-cell states, and immune cell distribution in breast cancer [36], as well as to assess the relationship between immune infiltration and treatment outcomes [37]. Despite these advances, several challenges remain. These include elucidating the role of ITH in limiting the efficacy of targeted therapies, devising strategies to identify drug-resistant subpopulations that contribute to poor long-term outcomes, and developing rational combination therapies that simultaneously target multiple signaling pathways [38]. Methodologically, single-cell suspensions prepared through enzymatic digestion disrupt the spatial context of cells within tissues. By contrast, spatial transcriptomics preserves spatial localization while capturing transcriptomic information, thereby providing more accurate insights into *in situ* gene expression, cellular functions, microenvironmental interactions, lineage tracing, and disease pathology [39,40].

In this study, we integrated spatial transcriptomics, single-cell sequencing, and bulk sequencing analyses to demonstrate that *LINC00467*, which displays spatial expression characteristics, is associated with breast cancer stemness, immunotherapy response, and drug sensitivity. Functional experiments further showed that silencing *LINC00467* inhibited the clonogenic capacity of breast cancer cells, potentially through regulation of the AKT pathway. AKT, also known as protein kinase B, is a central regulator of proliferation, metastasis, and invasion in breast cancer and is linked to multiple oncogenic markers and metastatic cascades [41]. Dysregulation of the PI3K/AKT signaling pathway occurs in up to 70% of breast cancers and is consistently associated with poor prognosis, underscoring AKT as an attractive therapeutic target [42]. LncRNAs with tissue-specific expression profiles may represent tissue specificity at different stages of breast cancer and thus may serve as biomarkers or therapeutic targets for breast cancer diagnosis and prognosis [43]. Several lncRNAs associated with breast cancer stemness have been identified, and once validated in prospective studies, they could enhance prediction accuracy and guide individualized treatment [44]. Notably, *LIN28* is an RNA-binding *protein* first identified in *C. elegans* and plays an important role in its development; in mammals, *LIN28* has two structural and functional homologs, *LIN28A* and *LIN28B*, and is highly expressed in various tumors, promoting cellular reprogramming and thus maintaining stem cell self-renewal and pluripotency [45–47]. Analysis of the TCGA BRCA cohort data revealed a significant positive correlation between *LINC00467* and *LIN28B*, while silencing *LINC00467* significantly inhibited *LIN28B* expression, suggesting that *LINC00467* may affect the stemness of breast cancer through *LIN28B*.

Due to the lack of direct empirical evidence. Computational screening via the RNAInter database revealed that *LINC00467* potentially interacts with multiple let-7 family miRNAs, including hsa-let-7b-5p, hsa-let-7e-5p, hsa-let-7g-5p, and hsa-let-7i-5p [24]. This is highly relevant as the let-7 family members are established negative regulators of *LIN28B*, a key driver of cellular stemness [31]. Thus, our bioinformatic finding suggests a potential mechanism where *LINC00467* may function as a molecular sponge for let-7 miRNAs, thereby attenuating their suppressive effect on *LIN28B*. The precise molecular mechanism underlying the regulation between *LINC00467* and the AKT pathway has yet to be experimentally validated. The direct interaction between *LINC00467* and the tumor suppressor miR-138-5p was confirmed by luciferase reporter and biotin RNA pull-down assays, demonstrating that *LINC00467* acts as a miRNA sponge to repress miR-138-5p expression. RIP assays further revealed that *LINC00467* directly binds to lin-28 homolog B (*LIN28B*), a key oncogene in breast cancer, leading to an increase in *LIN28B* protein levels [48]. However, the precise mechanism by which this interaction enhances *LIN28B* protein expression requires further investigation. The potential association between *LINC00467* and resistance to anti-PD-1 therapy remains an area of observational research. Conducting mechanistic explorations using co-culture models or multiplex immunohistochemistry techniques is of paramount importance. Supporting its clinical relevance, one study analyzing *LINC00467* expression in 113 normal breast tissues and 1091 breast cancer specimens reported significant overexpression in tumor tissues compared to normal controls [49]. Consistent with this, qRT-PCR analysis of 70 collected breast cancer samples and matched adjacent tissues demonstrated remarkable upregulation of *LINC00467* in cancerous samples. Further analysis indicated that *LINC00467* expression was positively correlated with tumor stage (*p* = 0.0164) and lymph node metastasis (*p* = 0.0248) in these patients [49]. Addressing these aspects will be a primary focus of subsequent research to fully delineate the functional and clinical significance of *LINC00467* in breast cancer.

Several limitations should be noted in this study. Spatial transcriptomic analysis was performed on only a single patient sample, limiting the generalizability of *LINC00467* expression patterns across breast cancer contexts. The observed inverse correlation between *LINC00467* expression and AKT inhibitor sensitivity relies solely on computational analyses of public datasets and requires functional validation using patient-derived organoids or isogenic cell lines. The precise mechanism through which *LINC00467* regulates AKT signaling remains unclear. Furthermore, this study lacks Western blot analysis of stemness markers (e.g., *ALDH*, *CD44*, *CD24*) in knockout vs. control cells and requires further validation via rescue experiments and RNA-protein interaction studies. The proposed role of *LINC00467* in cancer stemness also needs confirmation via stemness marker detection or *in vivo* xenograft models. Furthermore, its association with anti-PD-1 therapy resistance remains speculative and requires validation through co-culture systems or multiplex immunohistochemistry. Addressing these aspects will be a focus of subsequent analyses to fully elucidate the functional and clinical significance of *LINC00467* in breast cancer.

Our study indicated that elevated *LINC00467* expression is linked to cancer stemness traits in breast cancer. Functionally, *LINC00467* knockdown was shown to impair clonogenicity and attenuate AKT signaling pathway activation. Taken together, *LINC00467* represents a potential predictor of drug response in breast cancer.

## Data Availability

The data that support the findings of this study are available from the Corresponding Author, Junbin Yuan, upon reasonable request.
